# Outer casing structure design for the trisection turbine wheel burst of the air turbine starter

**DOI:** 10.1371/journal.pone.0310013

**Published:** 2024-09-27

**Authors:** Jin Tang, Yong Zhang, Ge Wang, Weidong Ma

**Affiliations:** AECC Hunan Aviation Powerplant Research Institute, Zhuzhou, China; Cyprus International University Faculty of Engineering: Uluslararasi Kibris Universitesi Muhendislik Fakultesi, TÜRKIYE

## Abstract

The aviation regulations mandate that high-energy rotor components must possesses adequate containment capabilities. Ensuring the containment of the turbine wheel of the air turbine starter is of paramount importance. In this paper, the design thickness of the containment ring was determined and the containment ring deformation was given. Based on the design thickness and deformation of the containment ring, an outer casing structure design method was proposed by using FEM. Then, two containment tests were conducted for different distances between the containment ring and outer casing to validate the outer casing structure design method. The errors of the containment ring deformation are smaller than 7.5%, and the experimental results of the containment process are in accordance with the simulation, validating correctness of the outer casing structure design method. The containment ring deformation rate with the design thickness T = 10 mm is 115%. A safety margin of 1.05 is designed by considering the uniformity of containment ring deformation and the containment ring assembly error. The results illustrate that the deformed containment ring does not damage the outer casing, when the inner diameter of the outer casing is designed as 1.2 times the outer diameter of the containment ring.

## 1. Introduction

Because of its lightweight construction and high power output, the air turbine starter (ATS) stands out as the optimal option for starting aircraft engines, finding extensive application within the aviation industry [1]. ATS operational velocity surpasses 60000rpm, indicating that the turbine wheel possesses significant kinetic energy [2, 3]. Hence, a significant aviation incident may ensue in the event of turbine wheel failure [4]. In 2007, ATS of the A330-300 encountered a turbine wheel fracture, leading to consequential damage to the integrated drive generator caused by the fragments of the broken wheel [5]. In 2013, ATS of the A330-302 encountered a turbine wheel fracture, resulting in damage to the oil pipeline from the fragments of the broken wheel [6]. Prior to 2007, a series of uncontained failures were reported in the ATS of CFM56 engines, causing harm to the fan cowl and engine [7]. Hence, ensuring the containment of the turbine wheel of ATS is of paramount importance.

In both America (FAR 25.1461) [8] and China (CCAR 25.1461) [9], aviation regulations mandate that high-energy rotor components must possesses adequate containment capabilities. As a result, numerous researchers have delved into exploring containment design method for turbine wheels. Hagg [10] conducted containment test for the quarter wheel burst to study the containment process. Gerstle [11] combined the finite difference numerical method with the large deflection theory, and developed an analytical simulation technology. By using the developed technology, the containment process of orthogonal braided fabric shield was simulated. Giard [12] investigated how the quantity of fractured wheel fragments correlates with the translational energy per fragment. The results show that trisection wheel bursts yield the highest translational energy per fragment. Frankenberger [13] experimentally investigated the containment capability of the containment ring for the Huey helicopter engine. Teng [14] simulated the containment process for the titanium turbine wheel piece obliquely impacting the aluminum panel. Stamper [15] proposed a prediction method of the containment process by using the commercial software ANSYS, which possessed good accuracy. Carney [16] conducted the containment test for a fan blade containment system. The results show that the alternate geometry meets the containment requirement and can make jet engine light. Li [17] numerically analysed the impact process of the trisection wheel fragment on the two different engine casing structures. Xuan [18] combined the simulation with test to investigate the impact process for the trisection wheel fragment on the engine casing. Winter [19] studied the containment process for the trisection wheel fragment impacting the compressor housing by simulation and experiment. There are many researches on the casing containment for aero-engine. However, there are few researches on the casing containment for ATS.

Bai [20] integrated containment simulation with test to investigate the impact process of the trisection wheel fragments on ATS containment ring. Zhang [21] proposed an optimal design method for the ATS containment ring, and validated by experiments. The research results demonstrate that the optimized containment ring can greatly decrease the containment ring thickness and containment ring weight. Chen [22] proposed an optimal design method of the ATS turbine wheel and the method is validated by experiments. The aforementioned researches concentrate on containment capabilities of containment ring for ATS. It should be noted that the turbine outer casing would be damaged by the deformation of the containment ring. However, there are few research on the outer casing structure design for ATS. Therefore, this paper proposed an outer casing structure design of ATS trisection turbine wheel burst, and gave two key design parameters which are containment ring thickness margin and outer casing inner diameter margin. The findings of this research can be applied to design ATS, offering significant engineering utility.

**Fig 1:**
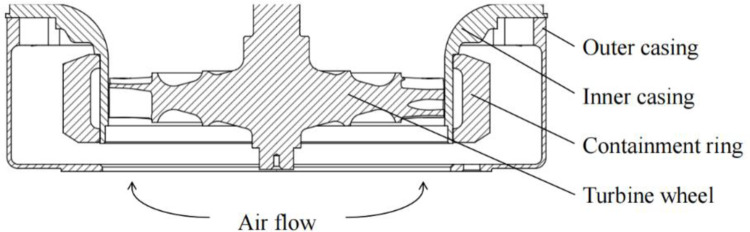
ATS containment structure.

**Fig 2:**
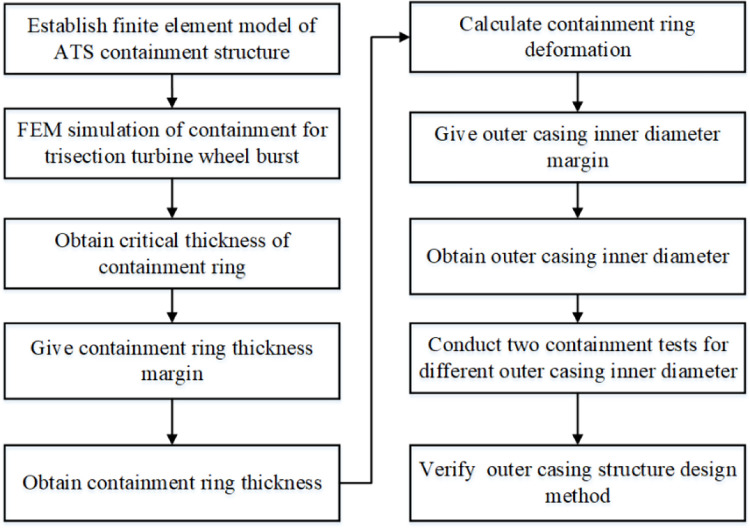
Flowchart of ATS outer casing structure design for the trisection turbine wheel burst.

Fig 1 is ATS containment structure, and Fig 2 is the flowchart of ATS outer casing structure design for the trisection turbine wheel burst. The rest of this paper can be outlined as follows. In Section 2, the critical thickness and maximum deformation of the containment ring were obtained, and the design thickness of containment ring was determined. In Section 3, an outer casing structure design method was proposed by using FEM. In Section 4, two containment tests were performed for different distances between the containment ring and outer casing to verify the outer casing structure design method. In Section 5, main conclusions are outlined.

## 2. Containment ring thickness design

The containment structure design for the trisection turbine wheel burst should meet two principal:(1) The wheel pieces do not penetrate the containment ring; (2) The deformed containment ring does not damage the turbine outer casing. Considering the first principle, the critical thickness of the containment ring should be obtained, and the design thickness of containment ring must be larger than the critical thickness. Considering the second principle, the maximum deformation of the containment ring should be obtained, and the inner diameter of the outer casing must be larger than the diameter of outer tangential circle of containment ring after deformation.

The impact energy reaches its peak when the wheel fractures into three identical pieces [12]. Therefore, the containment simulation of the trisection wheel burst are performed to obtain the critical thickness and maximum deformation of the containment ring. Then, the containment ring thickness is designed by the simulation results of the critical thickness and maximum deformation of the containment ring.

### 2.1. Material model

The turbine wheel and containment ring are adopted titanium alloy TC4 and nickel-based alloy GH3625, respectively. The Johnson-Cook (J-C) model [23, 24] is adopted for two components (as listed in Table 1), because J-C model accounts for the strain rate’s influence on material behaviour.

**Table 1:** J-C model material parameters of turbine wheel and containment ring.

Material	*A*(MPa)	*B*(MPa)	*n*	*C*	*M*	*D*_1_	*D*_2_	*D*_3_	*D*_4_	*D*_5_
TC4	1089	1083	0.93	0.014	1.1	-0.09	0.27	0.48	0.014	3.87
GH3625	447	1651	0.7	0.03	1.626	0.1	0.05	-4.028	0.171	-0.317

### 2.2. Finite element model and boundry conditions

**Fig 3:**
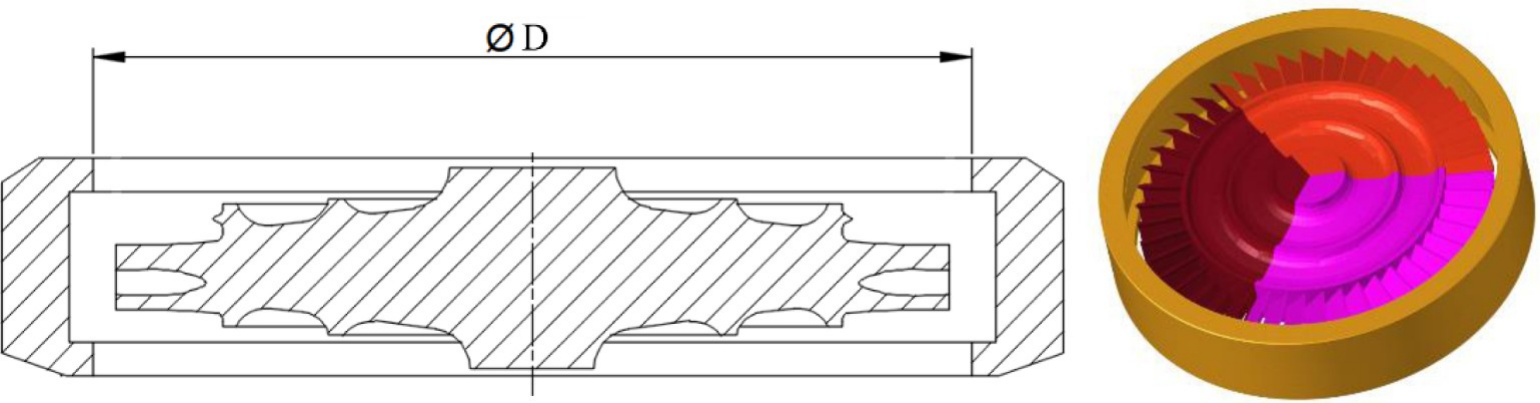
Geometric size and finite element model of turbine wheel and containment ring.

**Fig 4:**
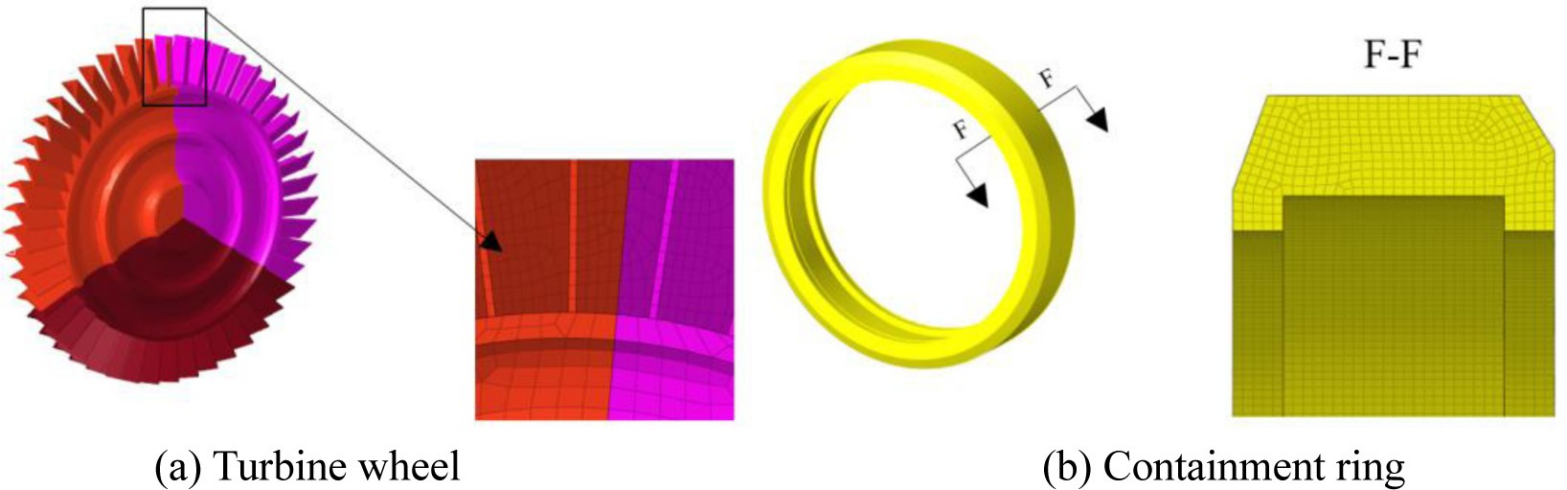
Mesh of turbine wheel and containment ring.

The software ANSYS LS-DYNA is used to simulate the containment process for the trisection turbine wheel burst of air turbine starters. Fig 3 are the geometric size and finite element model for the containment ring and turbine wheel. Fig 4 are two component meshes. To guarantee the precision of the computation, the hexahedral solid element is adopted for the two components. The mesh size of 1mm is adopted. The containment ring employs a free boundary condition that aligns with the actual situation.

**Fig 5:**
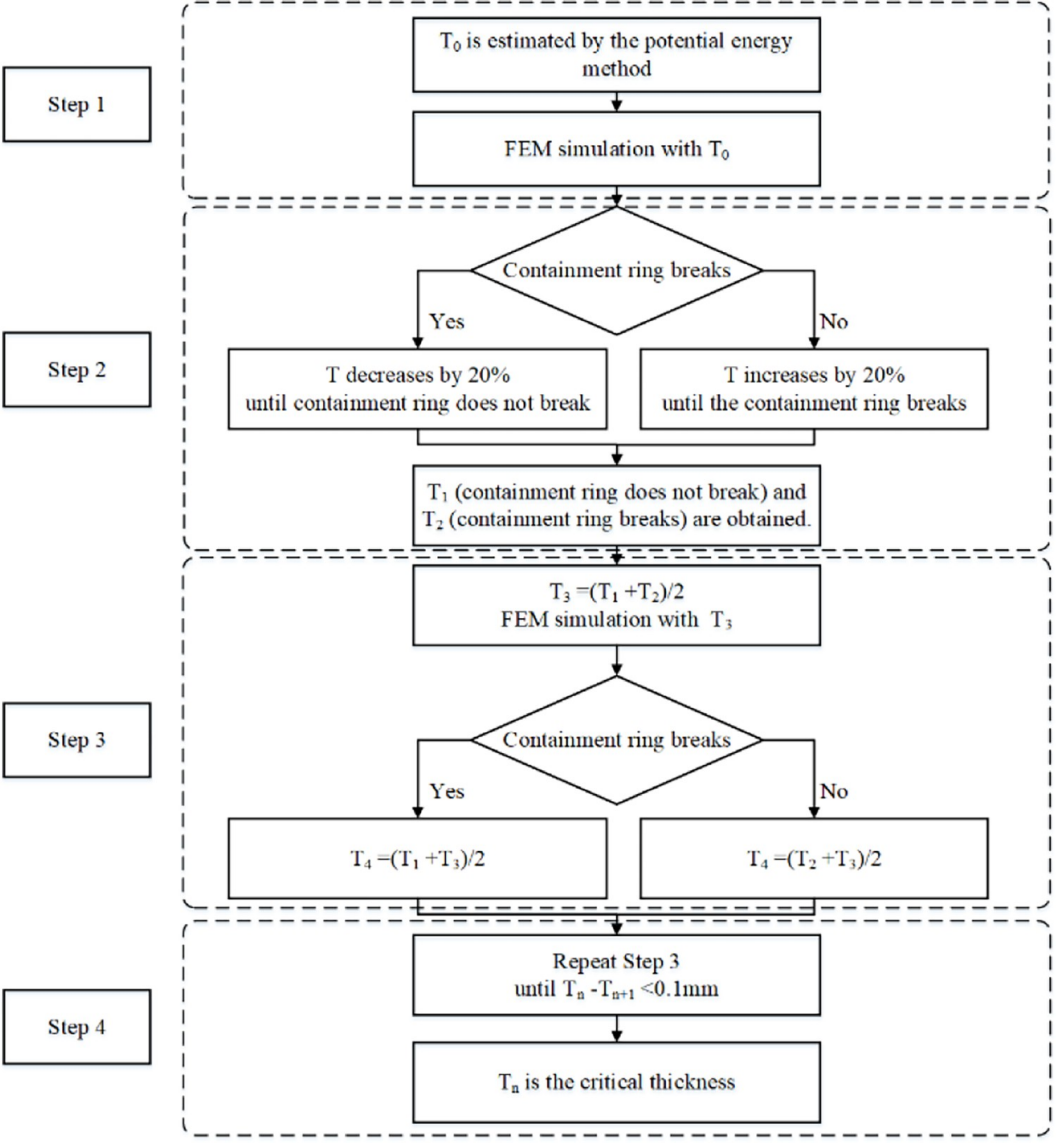
Critical thickness determination method.

T denotes the containment ring thickness. Fig 5 is the critical thickness determination method. Step 1: Initially, T0 is approximated using the potential energy method [12], following which the containment simulation is utilized with T_0_. Step 2: If the containment ring does not fracture, T decreases by 20% until the containment ring fractures; if the containment ring fractures, T increases by 20% until the containment ring does not fracture. Then two thicknesses of adjacent steps T_1_ (containment ring does not break) and T_2_ (containment ring breaks) are obtained. Step 3: The containment simulation is conducted with T_3_ = (T_1_+T_2_)/2. If the containment ring does not break, T_4_ = (T_2_+T_3_)/2; if the containment ring breaks, T_4_ = (T_1_+T_3_)/2. Step 4: Step 3 is repeated until two thicknesses of adjacent steps T_n_ (containment ring does not break) and T_n+1_ (containment ring breaks) is less than 0.1mm. Then T_n_ is the critical thickness.

When a clutch connection failure occurs, ATS might run at a speed where the turbine wheel spins freely without any load resistance on the output shaft, leading to potentially high rotational speeds. To prevent the turbine wheel from bursting under such free-run conditions, a safety margin of 1.15 is designed. This margin takes into account variations in material properties and manufacturing errors. For a specific ATS model, the free-run speed is determined to be n_0_ r/min, and the burst speed is accordingly set to n_1_ r/min, which is 1.15 times the free-run speed. Hence, the rotational speed n_1_ r/min is adopted.

### 2.3. Wheel burst containment simulation

**Fig 6:**
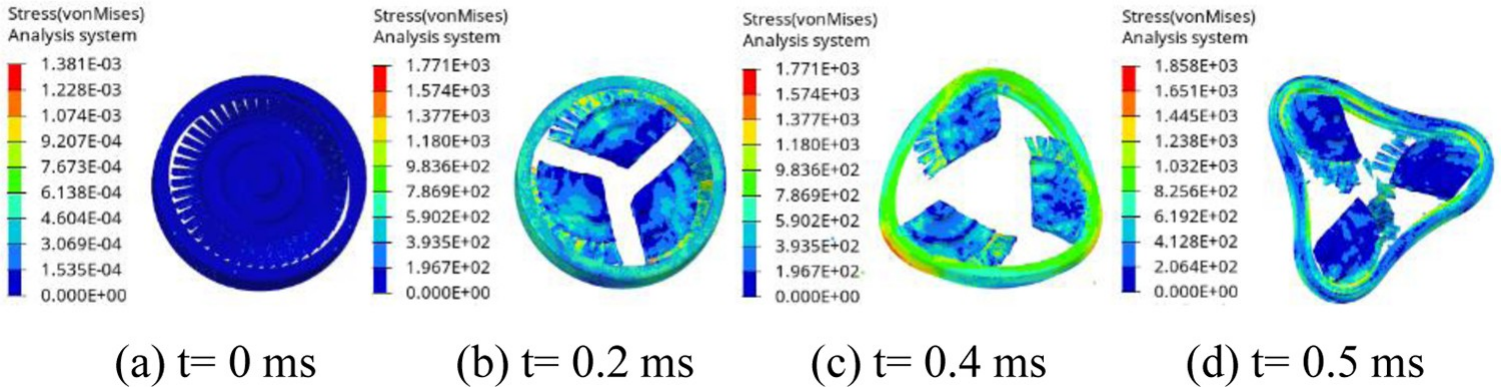
Process of the turbine wheel pieces impacting the containment ring with T = 8.35 mm.

Fig 6 is the impact process of the turbine wheel pieces on the containment ring at thicknesses of 8.35 mm. As seen in Fig 6, when t = 0.2 ms, the initial contact between the turbine wheel pieces and the containment ring occurs, leading to the containment ring deformation. By t = 0.4 ms, the containment ring undergoes plastic deformation, effectively absorbing the impact energy and gradually assuming a triangular shape. At t = 0.5 ms, the impact ceases, and the containment ring adopts a fully triangular deformation.

**Fig 7:**
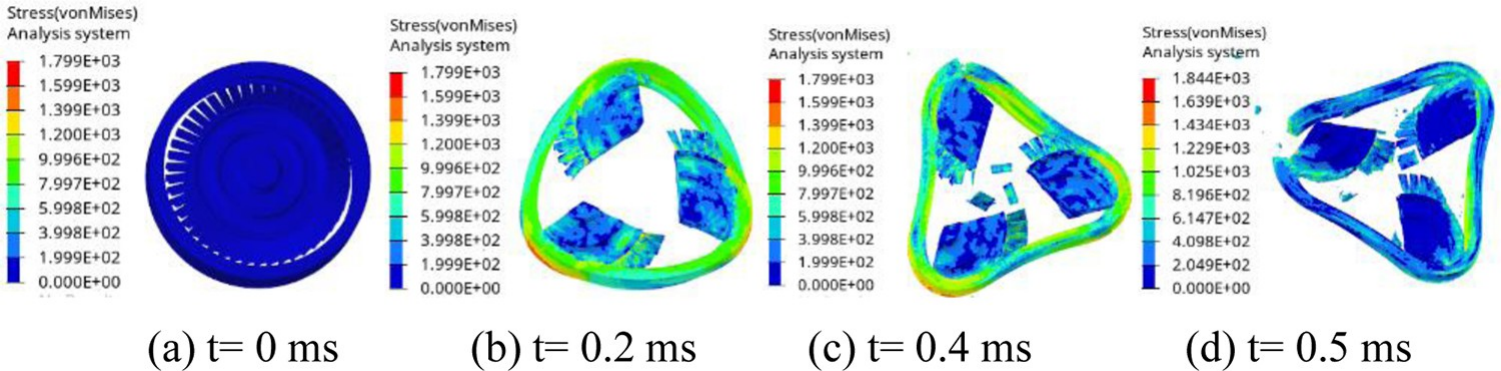
Process of the turbine wheel pieces impacting the containment ring with T = 8.28 mm.

Fig 7 is the impact process of the turbine wheel pieces on the containment ring at thicknesses of 8.28 mm. As seen in Fig 7, when t = 0.2 ms, the initial contact between the turbine wheel pieces and the containment ring occurs, leading to the containment ring deformation. By t = 0.4 ms, although the ring has fully deformed into a triangular shape, it has not absorbed all the impact energy, leading to the containment ring failure. At t = 0.5 ms, the containment ring fractures into three pieces. The simulations indicate that T = 8.35 mm is the critical containment ring thickness. T0 = 8.5 mm is thickness approximated by the potential energy method. The impact process with T0 is similar with that with T = 8.35 mm.

The containment ring deformation increase with the decrease of the containment ring thickness. Therefore, the maximum containment ring deformation is 127% corresponding to the critical containment ring thickness.

### 2.4. Determination of containment ring thickness

To prevent the penetration of turbine wheel pieces through the containment ring, a safety margin of 1.2 is designed. This margin accounts for variations in material properties, manufacturing inaccuracies, and the operational temperature of the containment ring. Therefore, T = 10 mm is the design thickness of the containment ring.

**Fig 8:**
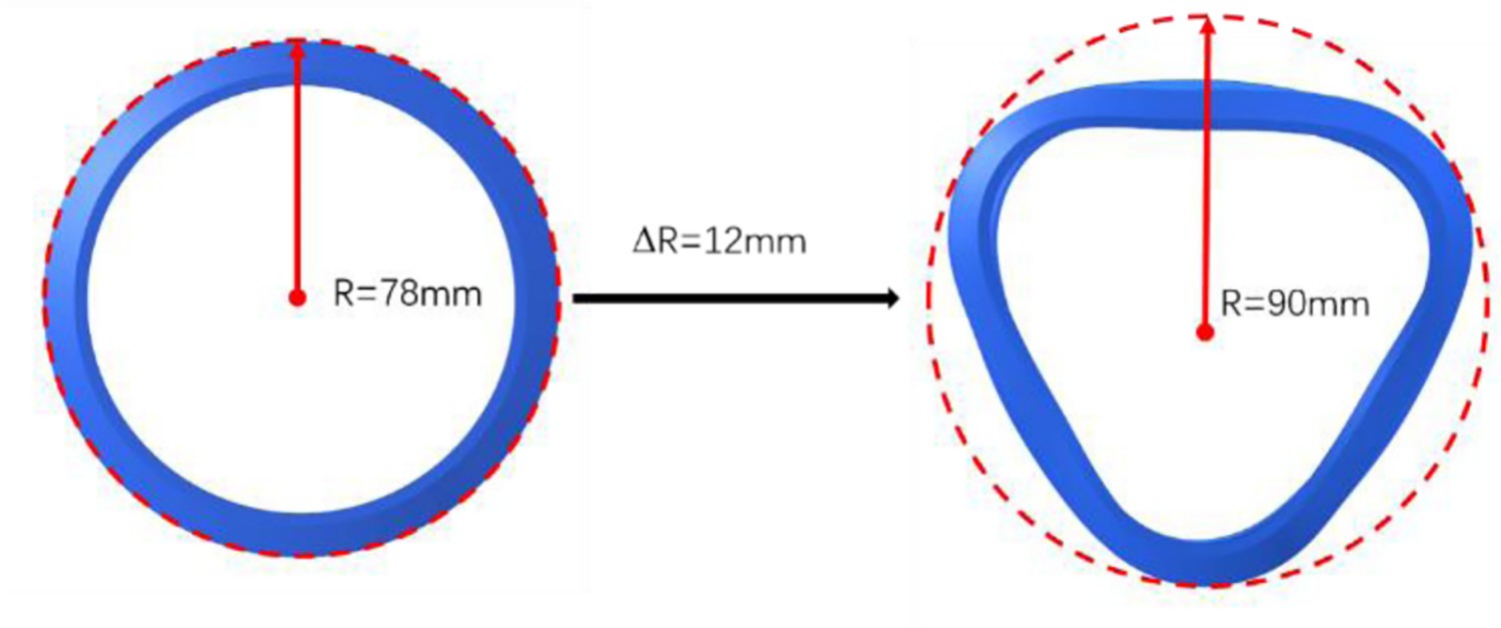
Deformation of the containment ring with T = 10 mm during the impact process.

The containment ring deformation with the design thickness T = 10 mm is shown in in Fig 8. During the impact process of the trisection turbine wheel burst, the containment ring radius changes from 78mm to 90mm. For the design thickness of the containment ring, the deformation of the containment ring is 12mm and deformation rate is 115.4%.

## 3. Outer casing structure design

Based on the design thickness and deformation of the containment ring, the outer casing structure is designed by the trisection wheel burst containment simulation.

### 3.1. Material model

The turbine wheel and outer casing are both adopted the titanium alloy TC4. The containment ring is adopted the nickel-based alloy GH3625. The J-C model parameters can be found in Table 1.

### 3.2. Finite element model and boundry conditions

**Fig 9:**
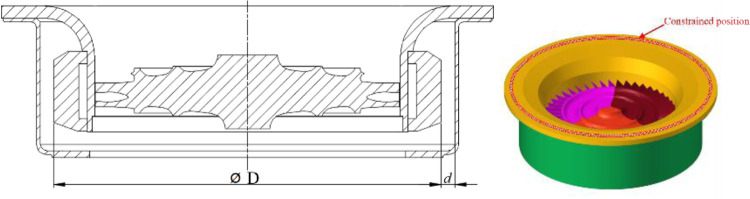
Geometric size and finite element model of four components.

**Fig 10:**
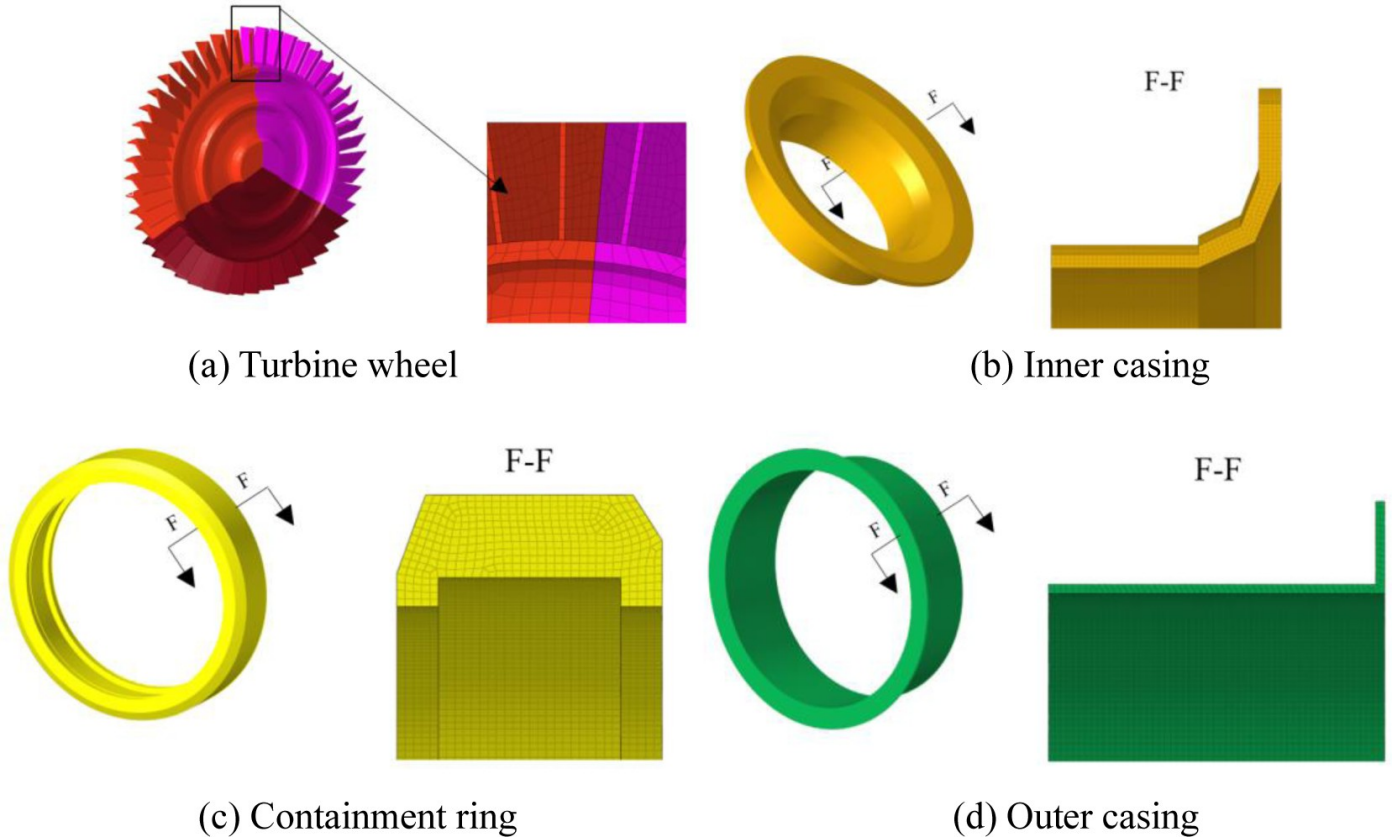
Mesh of four components.

The geometric size and finite element model of four components are shown in Fig 9. The mesh of four components are shown in Fig 10. To guarantee the precision of the computation, the hexahedral solid element is adopted for the two components. The mesh size of 1mm is adopted. The flange of the inner casing is tied with that of the outer casing, and constrained in displacement in all directions. The containment ring employs a free boundary condition that aligns with the actual situation.

Two distances between the containment ring and outer casing are selected to establish the model, as listed in Table 2. For a certain old type of ATS, the distance between the containment ring and outer casing is 5.5mm, so d1 = 5.5mm is selected. The containment ring deformation rate with the design thickness T = 10 mm is 115%. A safety margin of 1.05 is designed, taking into account the uniformity of containment ring deformation and the assembly error of containment ring. Hence, the inner diameter of the outer casing is designed as 1.15×1.05 = 1.2 times the outer diameter of the containment ring. d2 = 15.6mm is the design distance between the containment ring and outer casing.

**Table 2:** Two distances between the containment ring and outer casing.

Parameter	d1	d2
Value (mm)	5.5	15.6

### 3.3. Wheel burst containment simulation

**Fig 11:**
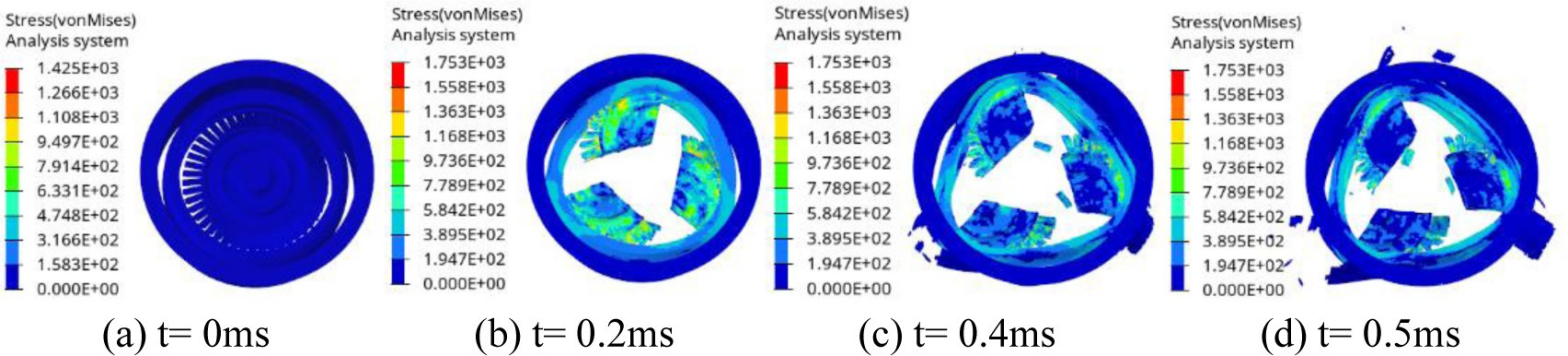
Process for the trisection wheel burst with d1 = 5.5mm.

**Fig 12:**
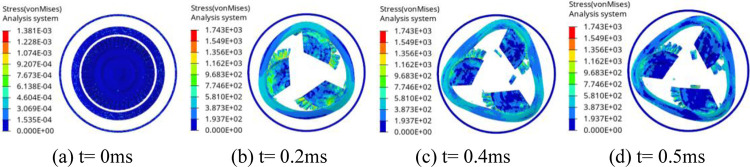
Process for the trisection wheel burst with d2 = 15.6mm.

Figs 11 and 12 are the process for the trisection wheel burst with d1 = 5.5mm and d2 = 15.6mm, respectively. As seen in Fig 11, when t = 0.2 ms, the initial contact between the turbine wheel pieces and the containment ring occurs, leading to the containment ring deformation. At this time, the containment ring does not contact the outer casing. By t = 0.4 ms, the containment ring undergoes plastic deformation, effectively absorbing the impact energy and gradually assuming a triangular shape. At this time, the deformation of the containment ring is larger than d1, and the containment ring impacts the outer casing, which makes the outer casing begin to damage. At t = 0.5 ms, the impact ceases, and the containment ring adopts a fully triangular deformation. At this time, the outer casing is completely destroyed by the impact of the containment ring.

As seen in Fig 12, when t = 0.2ms, the initial contact between the turbine wheel pieces and the containment ring occurs, leading to the containment ring deformation. By t = 0.4 ms, the containment ring undergoes plastic deformation, effectively absorbing the impact energy and gradually assuming a triangular shape. At this time, the deformation of the containment ring is smaller than d2, and the containment ring does not contact the outer casing. At t = 0.5 ms, the impact ceases, and the containment ring adopts a fully triangular deformation. At this time, the deformation of the containment ring is still smaller than d2, and the containment ring does not contact the outer casing in the whole containment process.

### 3.4. Determination of the distance between the containment ring and outer casing

To ensure the deformed containment ring does not damage the outer casing, a safety margin of 1.2 is designed, taking into account the uniformity of containment ring deformation and the assembly error of containment ring. And the simulation results illustrate that the containment ring does not contact the outer casing in the whole containment process. Therefore, the design distance between the containment ring and outer casing is 15.6mm.

## 4. Experimental verification of the outer casing structure design method

To validate the outer casing structure design method, two trisection wheel burst containment tests were conducted with d1 and d2, respectively.

### 4.1. Turbine wheel and containment ring

**Fig 13:**
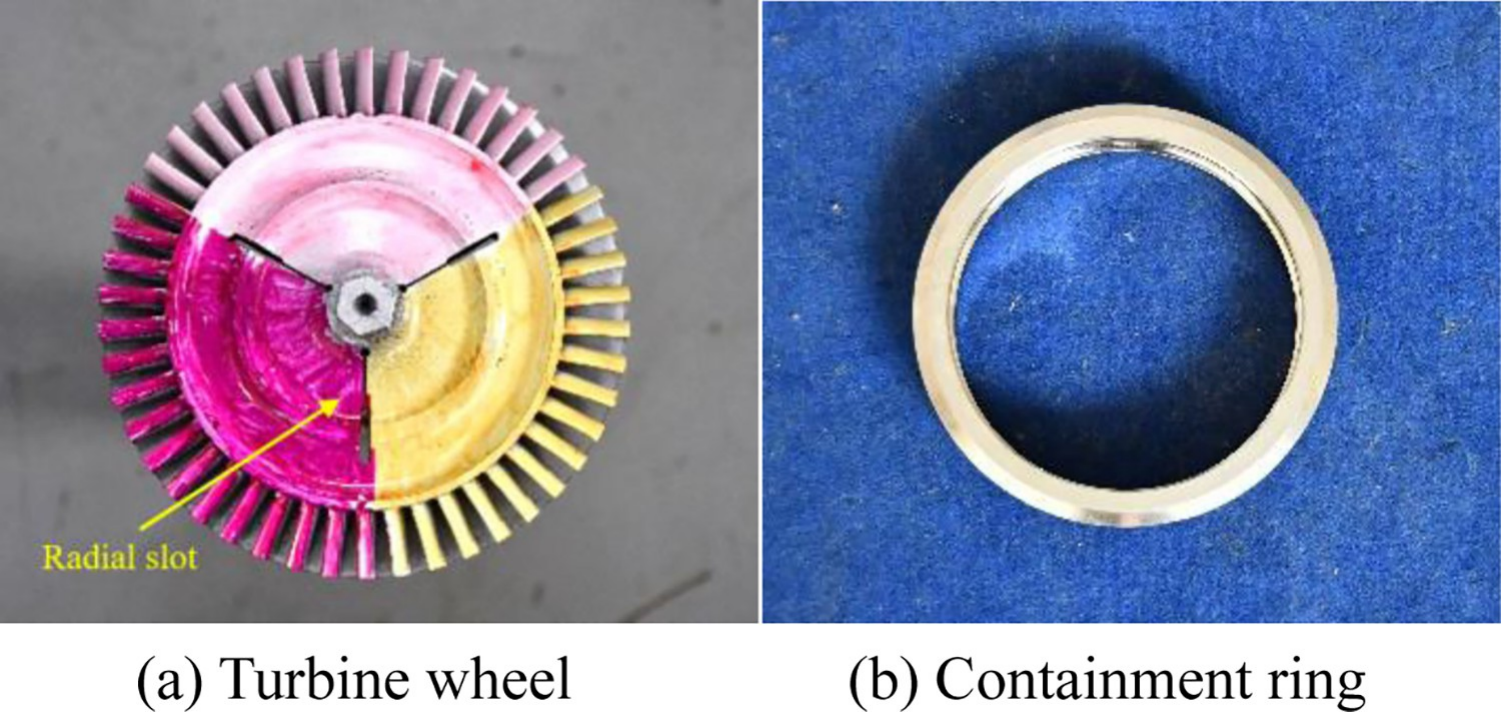
Practicality picture of the turbine wheel and containment ring.

Fig 13 is the practicality picture of the turbine wheel and containment ring. To realise the trisection wheel burst, the turbine wheel with three radial slots is adopted (Fig 13(a)).

### 4.2. Containment test

**Fig 14:**
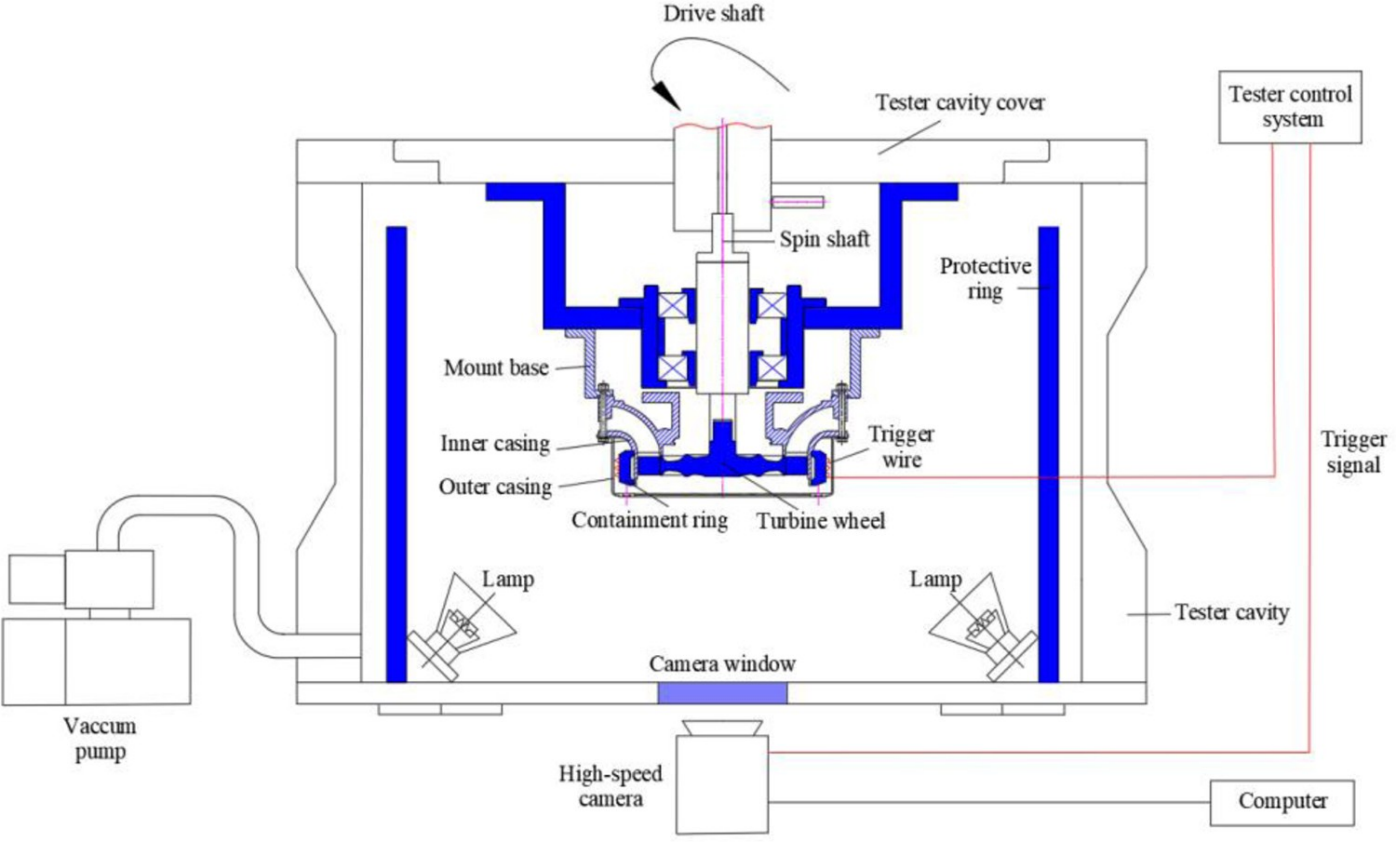
Containment test system.

**Fig 15:**
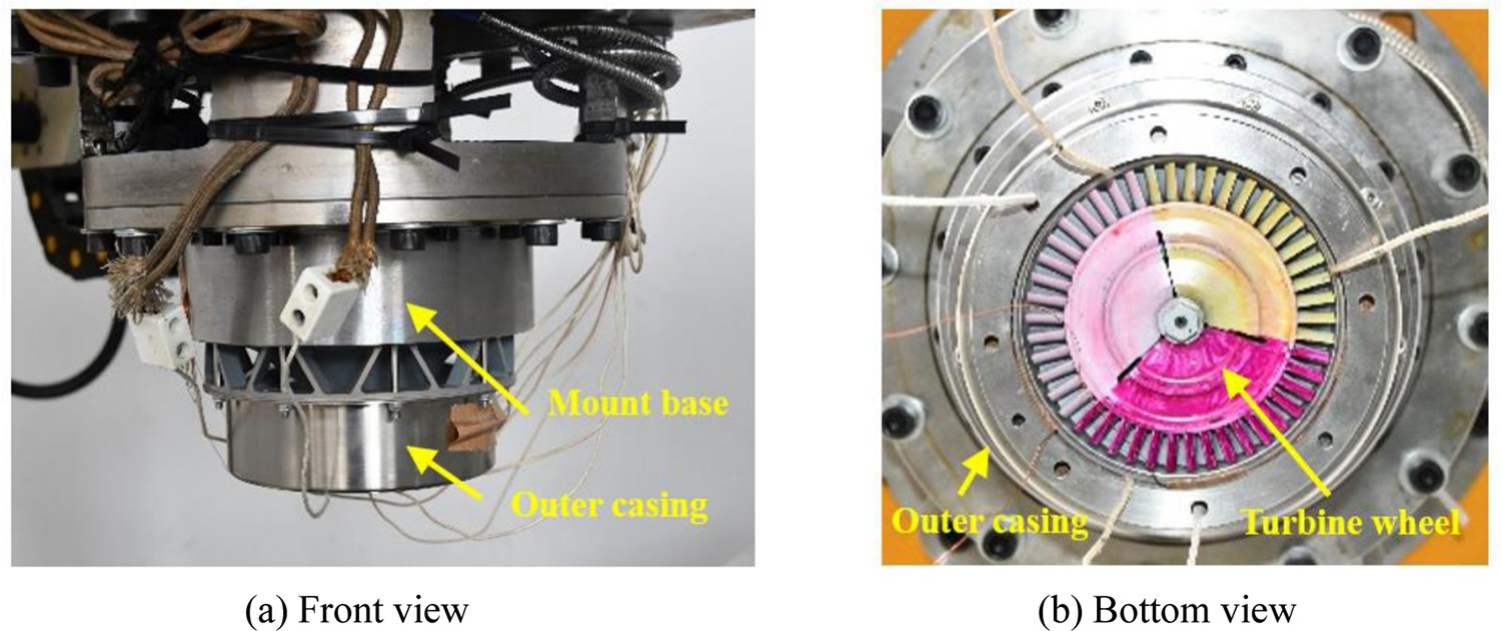
Practicality picture of containment test.

The containment tests are performed using a high-speed spin tester, and impact process of the trisection turbine wheel burst is recorded with a high-speed camera, as illustrated in Fig 14. The inner and outer casings are fastened together with bolts and are joined to the tester cavity cover via the mount base. The containment ring is located between the input and outer casings. Vacuum pumps are used to evacuate the tester cavity to 2 Torr to minimize air resistance. A trigger wire is affixed to the outer wall of the containment ring. When wheel pieces sever the trigger wire, a signal is dispatched to the control system to power down the drive motor. Simultaneously, the high-speed camera is activated to commence data capture. To guarantee a detailed recording of the impact process, the camera is set to a sampling rate of 51,000 frames per second (fps). Practicality picture of containment test is shown in Fig 15.

### 4.3. Comparison with experimental results

**Fig 16:**
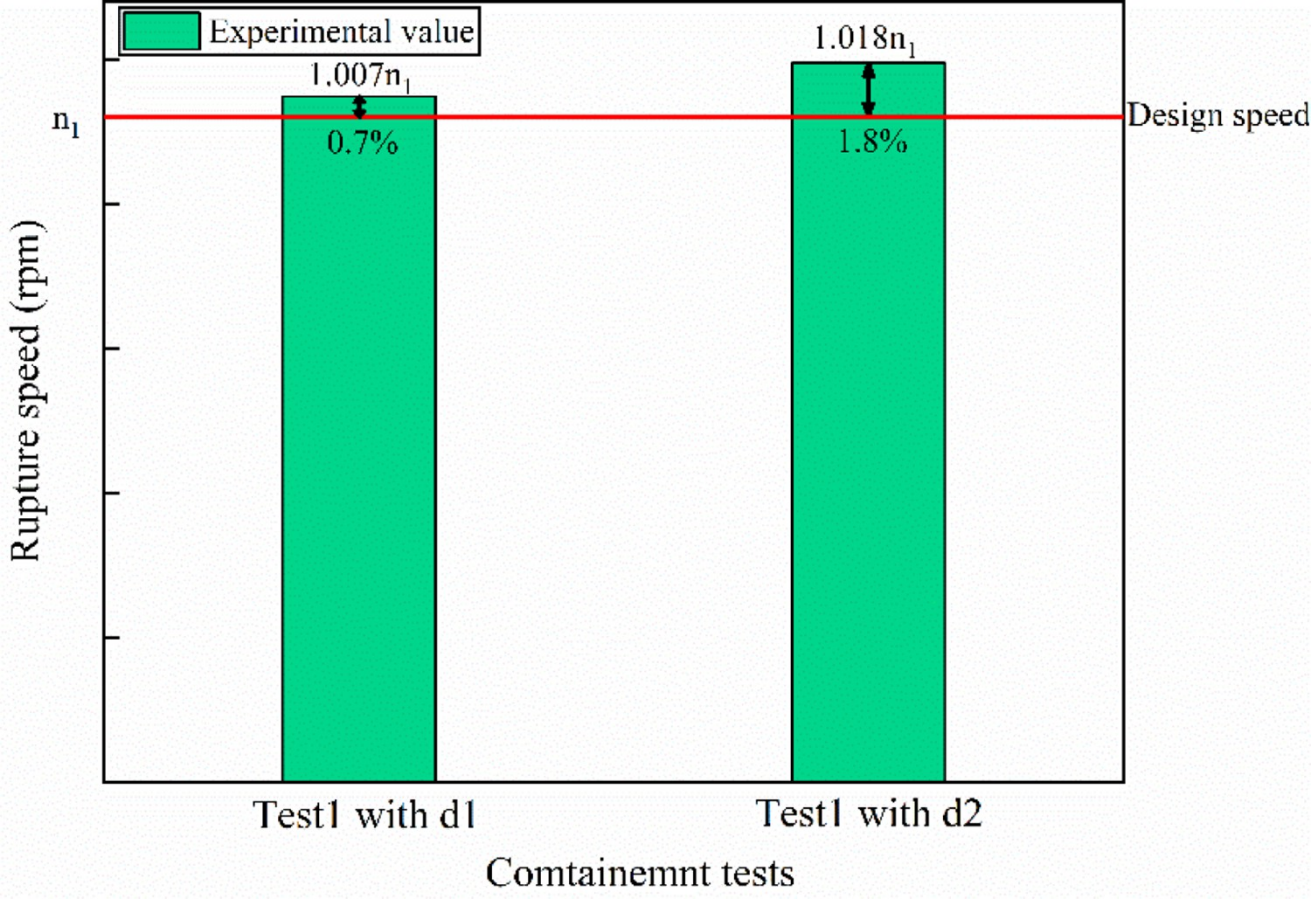
Burst speed for two containment tests.

Fig 16 is the burst speed of containment tests. As seen in Fig 16, the errors of two tests are less than 2%, demonstrating the reliability of the experimental results.

**Fig 17:**
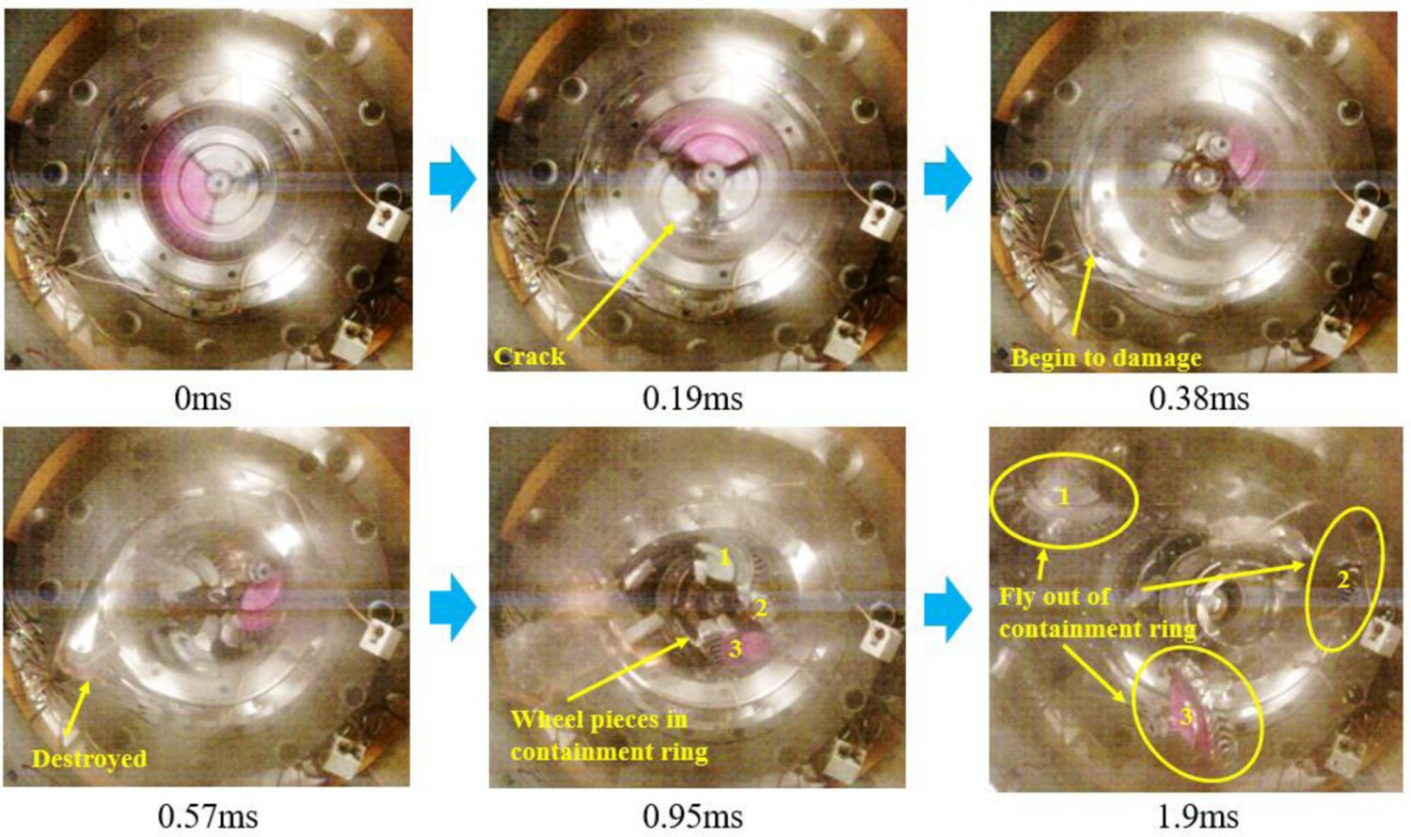
Process of the containment tests of the trisection wheel burst with d1 (5.5mm).

**Fig 18:**
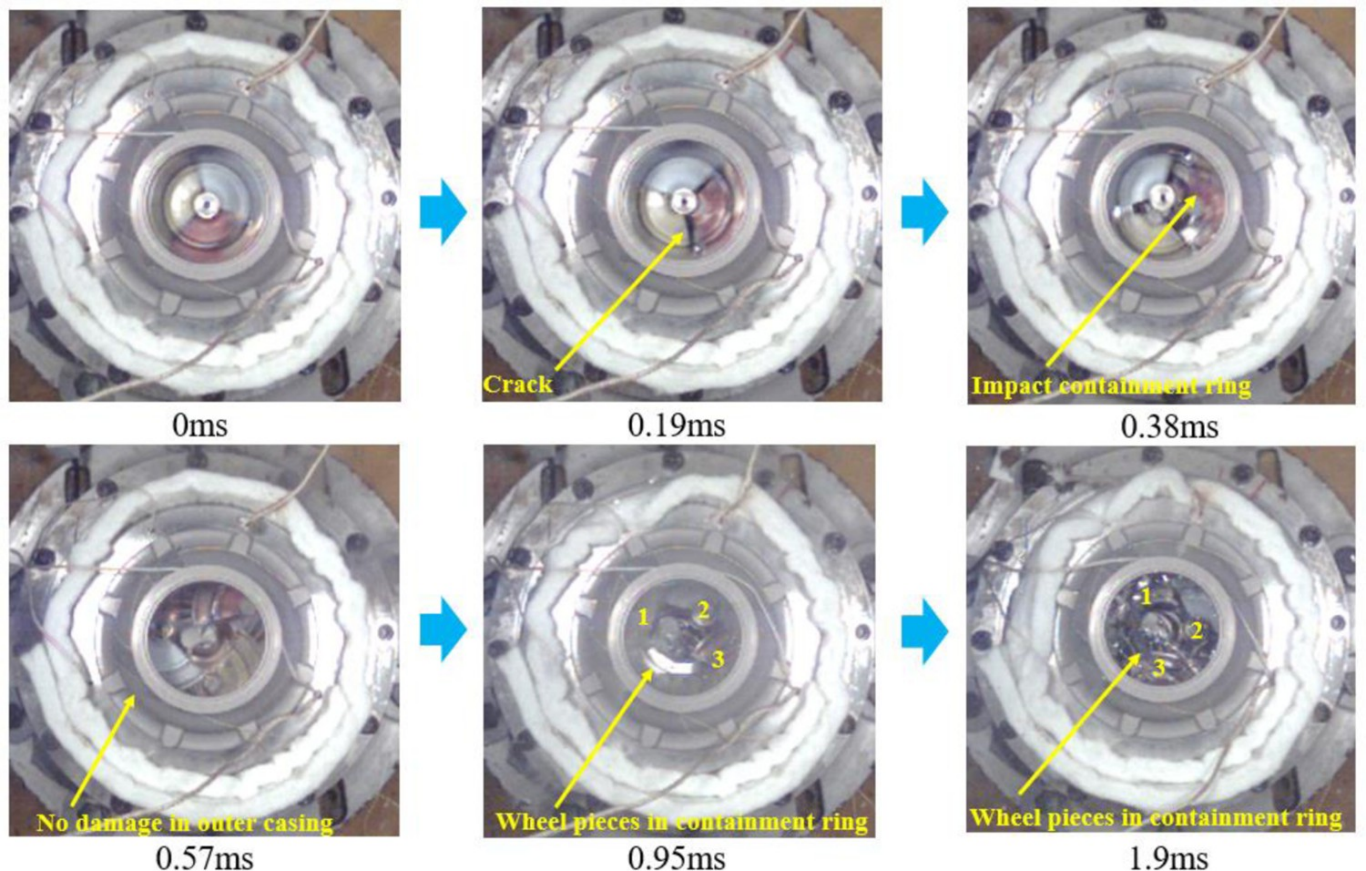
Process of the containment tests of the trisection wheel burst with d2 (15.6mm).

Figs 17 and 18 are the containment process for the trisection wheel burst with d1 and d2, respectively. Fig 17 illustrates that at t = 0.19ms, the turbine wheel fractures into three pieces, which break the inner casing and firstly contact the containment ring, and the containment ring starts to deform. At this time, the containment ring dose not contact the outer casing. By t = 0.38ms, the wheel pieces continue impacting the containment ring, which undergoes plastic deformation. The containment ring deformation is larger than d1, and the containment ring impacts the outer casing, which makes the outer casing begin to damage. At t = 0.57ms, the containment ring adopts a fully triangular deformation and the outer casing is completely destroyed by the impact of the containment ring. At t = 0.95ms, the containment ring begins to move in the radius direction without the constraints of the outer casing, but the wheel pieces are still located in the containment ring. When t = 1.9ms, the wheel pieces fly out of the containment ring.

Fig 18 illustrates that when t = 0.19ms, the turbine wheel fractures into three pieces, which break the inner casing and firstly contact the containment ring, and the containment ring starts to deform. By t = 0.38ms, the wheel pieces continue impacting the containment ring, which undergoes plastic deformation. The containment ring deformation is smaller than d2, and the containment ring does not contact the outer casing. At t = 0.57ms, the impact ceases, and the containment ring adopts a fully triangular deformation. At this time, the containment ring deformation is still smaller than d2, and the containment ring does not contact the outer casing in the whole containment process. At t = 0.95ms, the containment ring does not move in the radius direction with the constraints of the outer casing. When t = 1.9ms, the wheel pieces are still located in the containment ring, which means that the designed structure has sufficient containment. The experimental results align with the simulation, validating correctness of the outer casing structure design method.

**Fig 19:**
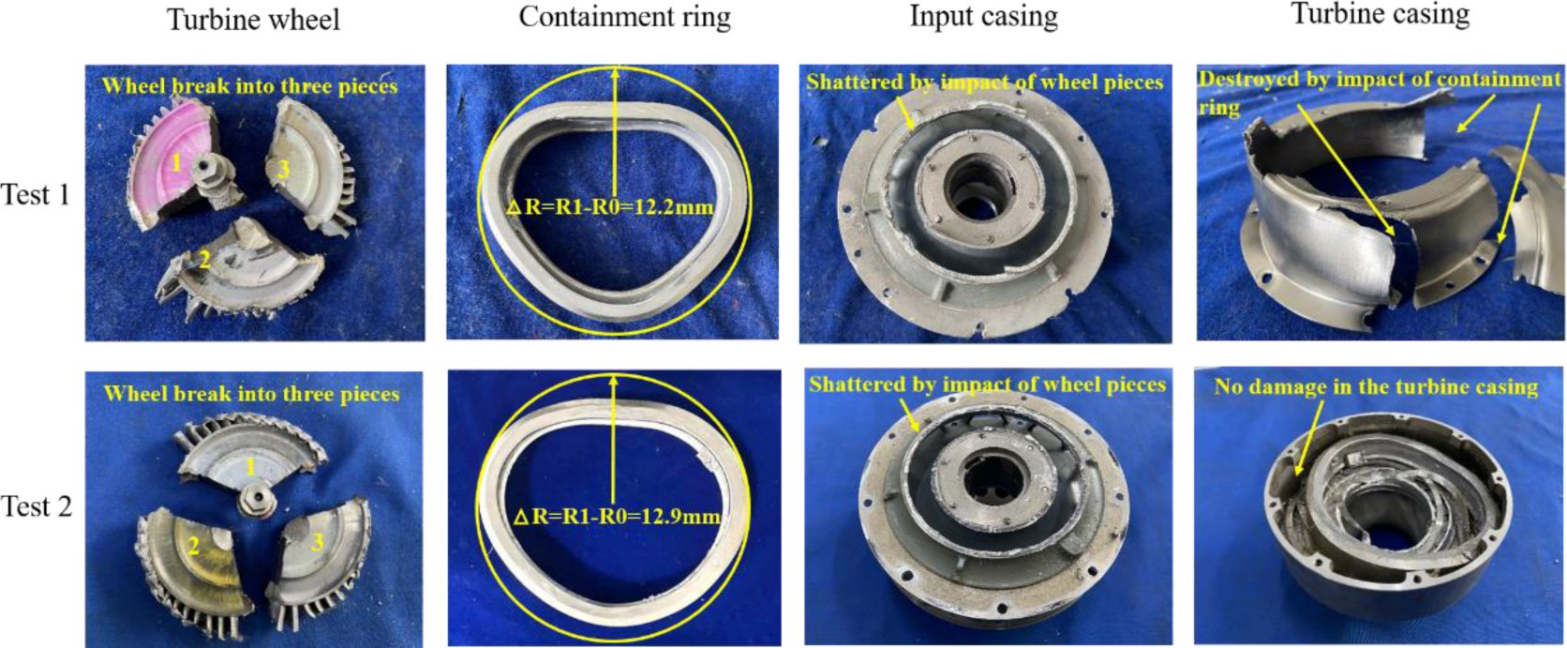
Damage condition of four components.

Fig 19 and Table 3 are the damage condition of four component for two containment tests. Fig 19 and Table 3 illustrate that compared with the design value, the errors of the containment ring deformation for test 1 and test 2 are 1.6% and 7.5%, respectively. The inner casing is shattered by the impact of the wheel pieces in both test 1 and 2. The outer casing is destroyed by the containment ring in test 1, while there exists no damage in the outer casing in test 2 because the deformation of the containment ring is smaller than d2. The experimental results align with the simulation, validating accuracy of the outer casing structure design method. d2 = 15.6mm should be adopted as the design value.

**Table 3:** Damage condition of two containment tests.

Parameter	Design value	Test 1	Test 2
Distance between containment ring and outer casing/mm	15.6	5.5	15.6
Containment ring deformation /mm	12.0	12.2	12.9
Inner casing status	Damage	Damage	Damage
Outer casing status	No damage	Damage	No damage

Combined with the simulation and experimental results, two key design parameters which are containment ring thickness margin and outer casing inner diameter margin are given. By using the containment ring thickness margin (1.2) and critical containment ring thickness (8.35mm), the containment ring thickness is obtained as 10mm. By using the outer casing inner diameter margin (1.05) and containment ring deformation (115%), the distance between the containment ring and outer casing is obtained as 15.6mm.

## 5. Conclusions

In this paper, the design thickness of the containment ring was determined and the containment ring deformation was given. Based on the design thickness and deformation of the containment ring, an outer casing structure design method was proposed by using FEM. Then, two containment tests were performed for different distances between the containment ring and outer casing to verify the outer casing structure design method. Main conclusions are outlined as follows:

1. The errors of the containment ring deformation are smaller than 7.5%, and the experimental results of the containment process are in accordance with the simulation, validating correctness of the outer casing structure design method.
2. The containment ring deformation increase with the decrease of the containment ring thickness. The critical thickness and maximum deformation rate of the containment ring are 8.35mm and 127%, respectively.
3. The containment ring deformation rate with the design thickness T = 10 mm is 115%. A safety margin of 1.05 is designed by considering the uniformity of containment ring deformation and the assembly error of containment ring. Therefore, for the trisection wheel burst, the deformed containment ring does not damage the outer casing, when the inner diameter of the outer casing is designed as 1.2 times the outer diameter of the containment ring.

To certify the ATS airworthiness, the vibration response of the outer casing and impact load transfer path should be studied. Therefore, the vibration response of the outer casing and impact load transfer path for ATS will be studied in our future research.
